# Lexical Access Restrictions after the Age of 80

**DOI:** 10.3390/brainsci13091343

**Published:** 2023-09-19

**Authors:** Carlos Rojas, Bernardo Riffo, Marilyn San Martín, Ernesto Guerra

**Affiliations:** 1 Department of Health Rehabilitation Sciences, Universidad del Bío-Bío, Chillán 3780000, Chile; msanmartin@ubiobio.cl; 2 Department of Spanish, Universidad de Concepción, Concepción 4030000, Chile; bernardo@udec.cl; 3 Center for Advanced Research in Education, Institute of Education (IE), Universidad de Chile, Santiago 8320000, Chile; ernesto.guerra@ciae.uchile.cl

**Keywords:** aging, fourth age, cognition, language, lexical access

## Abstract

Background: During the fourth age (80+ years), cognitive difficulties increase. Although language seems to resist the advancement of age, an older person without pathological developments in cognition may exhibit deficits in lexical access. This study examines the restrictions on lexical access in people aged 80 and older in word recognition and retrieval modalities through four lexical tasks. Method: The effect of aging on response time and accuracy was measured using recognition (lexical decision/naming/priming) and retrieval (picture naming) tasks. A fourth age group (>80) and two third age groups (60–69/70–79) were compared according to lexical access modality and type of task employed through linear regression models. Results: People aged 80 and older exhibit a strong lexical access constraint, as they are slower and less accurate in recognizing and retrieving words than both third age groups. These restrictions are more profound for the word retrieval modality, especially in the picture naming task. Conclusion: Impaired fluid intelligence and internode transmission deficits during advanced aging could further reduce the ability to recognize and/or retrieve words, having an impact on access speed and accuracy. Furthermore, the idea that crystallized intelligence could strengthen the accuracy of lexical access during aging is supported, specifically in word recognition modality.

## 1. Introduction

The changes associated with aging and increased longevity in modern societies have resulted in two distinctive groups: the third age (60–79 years) and the fourth age (>80 years [1]). It is during the fourth age when executive and instrumental difficulties increase, affecting the cognitive functioning of older individuals more severely [2,3]. These changes significantly impact logical problem-solving skills, operational abilities, and processing speed (also known as fluid intelligence [4]).

Similarly, older individuals see an increase in their reserves of experience, knowledge, and vocabulary (i.e., crystallized intelligence), allowing them to improve their cognitive performance to some extent [5]. There is evidence that people aged 80 and older retain their crystallized intelligence but see a progressive decline in fluid intelligence [6], eventually reaching a basal level of functioning, which results in a generalized cognitive decline [2,7]. Thus, the fourth age presents a mixed functional configuration, with some aspects showing evident impairment (such as working memory and executive functions), while other capacities appear better preserved, including language [8].

Although language seems to resist the advancement of age, an older person without pathological developments in cognition may exhibit deficits in comprehension and production [9,10]. However, this decline is asymmetrical, as production processes are more vulnerable to aging than comprehension processes, a product of the latter aspect presenting further benefits in a communicative context [9,11]. In lexical access, particularly, dissimilar behaviors are also observed in older people. Specifically, they exhibit an incipient deficit in word recognition at the comprehension level [8,12], accompanied by the early and progressive appearance of significant difficulties in lexical retrieval and production [13,14,15,16], where this latter modality is identified as the most frequent linguistic constraint during aging [9,10,11,13,17].

Considering this background, it is reasonable to assume that recognizing or retrieving words after the age of 80 may be a complex task and that deficits should increase due to the cognitive decline associated with advanced aging [3]. This complexity arises from the multiple levels involved in lexical access, such as the activation, selection, and inhibition of competitors [9,14,18]. In this context, it is worth asking: How does the cognitive deficit of old age impact lexical access after age 80? How severe is the restriction in word recognition and retrieval?

Studies using chronometric reading techniques have found that older individuals are slower than younger people (longer response time, RT) but are nearly equally accurate at word recognition tasks, both in isolation and in the context of sentences [8,19]. Additionally, studies using lexical decision tasks (LDT) and priming have also confirmed that older individuals take longer (higher RT) but make a similar number of errors as younger people in recognizing words and distinguishing them from pseudowords [20]. As for the fourth age, specific studies have revealed that the RT needed to recognize isolated words increases significantly compared to the third age, whereas the accuracy remains stable for low cognitive load tasks such as naming and priming [12,21].

Regarding lexical retrieval, studies using free word production, picture naming, lexical availability, and others [13,14] have revealed that older individuals have evident difficulties in word production. In general, these studies have found a significant increase in RT and a reduction in accuracy compared to younger people [11,14,18]. The difficulties in word retrieval are more evident when older individuals need to find the appropriate word to name certain pictures, access proper names, label a given definition [13], name visually presented objects [18], and consecutively name items within the same semantic field [13,17]. Moreover, older individuals often exhibit the “tip of the tongue” phenomenon [11,14,18], which means that it takes them longer than normal to access the phonological form of a specific lexical representation, or they simply cannot do so, even though they have a clear sensation of knowing the word and having it “on the tip of the tongue” (since they can access the semantic reference). In turn, Rojas, Riffo, and Guerra [22] found that people after age 80 exhibited longer RT in word retrieval tasks and a reduced number of retrieved words, as well as a significantly reduced lexical availability index.

Lexical access restrictions, especially a word production deficit, appear frequently from age 50 and become common after 70 [23], where picture naming tasks become more complex due to the early impairment of access to the phonological form of the selected lemma [14,15]. Thus, everything suggests that lexical retrieval deficits increase with age [9] and could eventually affect communication and social interaction in advanced aging by creating a sense of linguistic incompetence [2]. On the other hand, lexical recognition tasks seem to always exhibit a better performance, especially in early old age when it exhibits a very low error rate [24]. From this viewpoint, new questions arise: Do retrieval deficits continue to predominate over recognition deficits during the fourth age, and does the picture naming task generate greater processing constraints in RT and accuracy in advanced aging compared to LDT, naming, or priming?

Moreover, several studies on lexical access have shown that some lexical variables play a facilitating or inhibiting role in the recognition and/or retrieval processes [25]. For example, high-frequency words, compared to low-frequency words, reduce the RT and increase the accuracy in lexical access tasks, since they have greater semantic representation and familiarity [26]. They also form more interconnected networks between sub-lexical units, thereby having a greater potential to be recognized, which facilitates their activation and subsequent selection [25]. The lexical frequency has shown stable behavior throughout the life cycle and has been extensively studied both in young people and in the early stages of aging but not in the fourth age. Thus, it is interesting to explore whether the effect of this variable remains stability in recognition and retrieval tasks after the age of 80, a stage in which cognitive decline marks the evolutionary course of this group.

### The Present Study

The aim of this research is to explore the restrictions exhibited by lexical access in people aged 80 and older in both word recognition (comprehension) and word retrieval (production) modalities, as well as the type of task employed. The effects of aging on response time (RT) and accuracy were evaluated using recognition tasks (lexical decision task (LDT), naming, and priming) and a word retrieval task (picture naming) in a fourth age group (>80 years old) compared to two third age groups (60–69 and 70–79 years old). It was predicted that the fourth age group, compared with their younger peers (third age groups), would have greater lexical access restrictions. Additionally, the deficit in fluid intelligence during the fourth age would further impair word retrieval. This would be most evident in the picture naming task.

## 2. Materials and Methods

### 2.1. Participants

A total of 90 monolingual older adults participated in the study voluntarily. The sample was divided into three age groups of 30 individuals each. Group 1: 60–69 years (M = 65.73 years, SD = 2.99; M = 13.00 years of schooling, SD = 1.23), group 2: 70–79 years (M = 74.00 years, SD = 2.89; M = 13.13 years of schooling, SD = 1.81), and group 3: 80–92 years (M = 82.53 years, SD = 3.10; M = 13.03 years of schooling, SD = 1.71). The first two groups represented the third age group, and the last one represented the fourth age group. All participants signed an informed consent form approved by the university’s ethics committee sponsoring the research. The inclusion criteria were age 60 years or older; 8 years or more of schooling; self-reported active aging (physical, social, and mental well-being); normal (or corrected) vision and hearing; living in an urban area; and performing the tasks within 2 months. The exclusion criteria were presenting cerebrovascular or neurodegenerative disease; depression or psychiatric disease; or risk scores in any of the applied screening tests: <21 points in the Montreal Cognitive Assessment (MoCA [27]), >11 points in Geriatric Scale of Depression-15 [28,29], and <4 points in the reading comprehension Boston subtest [30]. In addition, the participants’ medical care records had to be up to date, indicating the optimal cognitive performance.

Initially, participants were contacted through a connection between the university and three local senior citizens’ clubs. The study’s objectives, benefits, and risks were explained to the authorities of the organizations, and subsequently, those willing to collaborate were assessed for correct cognitive, emotional, and reading performances using the MoCA test, GSD-15 Scale, and Boston Reading subtest, respectively. Finally, the selected persons were invited to the Language and Cognition Laboratory of the sponsoring university to perform the lexical tasks.

### 2.2. Materials and Design

In this study, 28,800 RT and accuracy of the available data corresponding to an equal number of words used in the LDT, naming, priming, and picture naming experiments we analyzed.

For LDT, a 2 × 2 × 3 design was constructed, considering the factors of lexical frequency (high = absolute frequency equal to or higher than 14.0 (number of times the word appears in the Spanish Lexical Database corpus divided by the total word count of the corpus multiplied by one million), range 418.5 (day)–14.03 (shoes), average value of 96,56/low = absolute frequency between 0.60 and 1.57, range 0.61 (clone)–1.57 (champagne), average value of 0.95), according to the Spanish Lexical Database (https://www.bcbl.eu/databases/espal/, accessed on 9 September 2019); imaginability (high, i.e., stimuli that evoke vivid mental images = scores between 5 and 7 according to a normative study/low, i.e., stimuli that do not evoke strong or vivid mental images = score between 1 to 3); and age group (60–69, 70–79, and 80+ years). It incorporated a total of 150 trials (60 words analyzed, 60 pseudowords, and 30 filler trials).

For naming, a 2 × 2 × 3 design was constructed, considering the factors of lexical frequency (high/low equal to LDT); positional syllable frequency of the first syllable (high, i.e., refers to words that contain syllable structures that are commonly used in a particular language/low, i.e., refers to words that contain syllable structures that are less commonly used in a particular language), according to Álvarez et al. [31], and age group (60–69, 70–79, and 80+ years), with a total of 150 trials (60 words analyzed, 60 pseudowords, and 30 filler trials).

The priming task incorporated a 2 × 3 × 3 design, considering the factors of lexical frequency (high/low equal to LDT); prime type ((1) semantic, i.e., related in terms of meaning, or associative, i.e., they imply an associative relationship between stimuli/(2) ortho-phonologic, i.e., the stimuli share orthographic and phonological similarities; according to a normative study); and age group (60–69, 70–79, and 80+ years), with a total of 100 trials (80 words analyzed and 20 filler trials).

Finally, picture naming incorporated a 2 × 3 × 3 design, considering the factors of lexical frequency (high/low equal to LDT); syllable length of the word (bisyllabic/trisyllabic/tetrasyllabic); and age group (60–69, 70–79, and 80+ years), with a total of 150 trials (120 words analyzed and 30 filler trials). For further details on the words used and their lexical frequency, please refer to Supplementary Materials 1 and 2.

### 2.3. Procedure

The experiments were performed in random order, in a lighted and soundproofed room. Stimuli were presented in the center of a 15.6-inch computer screen using E-Prime 3.0 software. In the LDT, participants were instructed to decide whether the stimulus presented was a word or not. For this, they had to answer “yes” in case of words and “no” in case of pseudowords. Each trial started with a fixation point (a star in the center of the screen) for 1000 ms, followed by the target stimulus, written in capital letters and presented randomly. Once a verbal response was generated, a 1000 ms feedback was given, and the next trial began. If there was no response after 10 s, the examiner, by pressing key 1 on the keyboard, recorded the response (computed as invalid) and allowed the passage to the next trial. Participants were instructed to respond quickly when the stimulus appeared. The computer, using the vocal key (the Chronos response box driven by E-prime 3.0 software was used), monitored and recorded the time elapsed from stimulus presentation until the participant responded orally. This recording made it possible, a posteriori, to determine the successes and errors in the responses and thus calculate their accuracy. The experiment was carried out in three blocks, and its application lasted between 20 and 25 min. For the naming task, an identical configuration was used as for the LDT, with the difference that, this time, the participants had to read aloud each of the words and pseudowords presented as quickly as possible and without making errors. The task was also divided into two blocks and lasted approximately 20 min. The priming task, on the other hand, presented a similar procedure to that set up for the naming, except that this task incorporated the presentation of a prime stimulus for 1000 ms prior to the appearance of the target word. Participants were instructed to first read primes silently and, subsequently, to read the target words aloud. The task was presented in 3 blocks and lasted 20–25 min. Finally, in picture naming, the participants had to name each of the pictures as quickly as possible and without making errors. Each trial began with a fixation point of 500 ms, followed by an attentional click for 1000 ms, which was followed by the image. Once the response was obtained, a 1000 ms feedback was presented, and the next trial began. The task was presented in 3 blocks and lasted 30–35 min. The details of the experimental trials for each task are presented in Supplementary Materials 3.

### 2.4. Data Analysis

The number of correct and incorrect responses was recorded for each task. Trials with very long (>6000 ms) or very short (<200 ms) latencies, or responses resulting from involuntary activation of the vocal key, were considered invalid, following the criteria of Ratcliff et al. [21]. Invalid trials represented 4.42% in LDT, 2.28% in naming, 3.44% in priming, and 5.53% in picture naming. After calculating the basic statistics for each experiment (mean and SD), a descriptive analysis was performed for response time (RT) and accuracy by age group and lexical task. The RT data were log-transformed to fit a normal distribution, while the accuracy data were fitted to a binomial distribution. Cross-mixed effects regression models were used for inferential statistical analysis, comparing lexical modality and task type. Linear models were run for RT, and generalized linear models were used for accuracy. The lme4 [32] and lmerTest packages [33] of R statistical software were used. These models allow accommodating the intrinsic variability by participant and item in a single regression, without the need to add more data.

Two models were run for the analysis of RT and accuracy for the modality variable, which included as predictors three factors: age group (60–69, 70–79, and 80+ years); modality (recognition: TDL/naming/priming/; retrieval: picture naming); and lexical frequency (high/low). The overall average of the fourth age group served as the intercept of the regression, allowing for comparisons with the third age groups and the predictors. Two other models were applied for analysis by experiment type, including the factors age group, experimental task (LDT/naming/priming/picture naming), and lexical frequency as predictors. The fourth age group and picture naming were used as a comparison intercept, allowing for a comparison of the average of this group and the technique applied with the various predictors. All models incorporated interactions between variables and included random intercepts at the participant and item levels, as well as random slopes for the experimental variables.

## 3. Results

### 3.1. Descriptive Results

The descriptive analysis of variables RT (ms) and accuracy (%) for each age group, applied task, and lexical frequency (Table 1) shows that RT increases progressively with age and in each of the applied tasks. On average, picture naming and LDT tasks generate slower responses compared to naming and priming tasks. While the accuracy is slightly lower for fourth age individuals in all tasks, the naming and priming tasks exhibit a ceiling effect with a hit rate above 97% throughout aging. In contrast, the picture naming task shows the lowest accuracy, counter to the rest of the tasks, in both the third and fourth age groups. High-frequency words obtained lower RT and higher accuracy in all age groups and in each of the tasks performed (see Table 1).

### 3.2. Results by Lexical Modality

**Figure 1:**
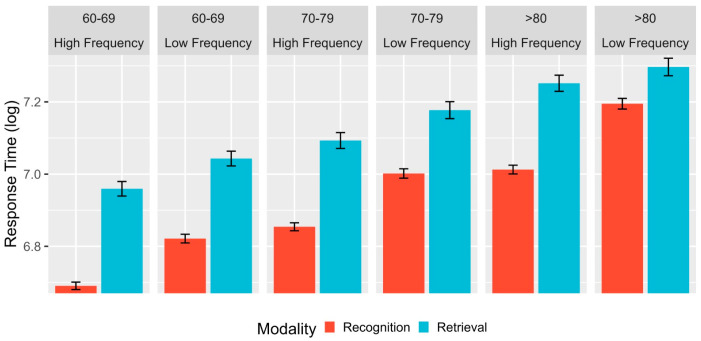
Mean log-transformed RT as a function of age range (80+, vs. 60–69 vs. 70–79), modality (recognition vs. retrieval), and lexical frequency (high vs. low). Error bars represent within-subject adjusted 95% confidence intervals.

Regarding the comparative analysis between the third and fourth age groups in terms of lexical access modality, the linear mixed-effects regression (Table 2) revealed a significant effect of age on response time (RT). Specifically, fourth age participants had slower responses compared to their peers aged 60–69 (β = −0.329, se = 0.033, t = −10.098, *p* < 0.00) and 70–79 years (β = −0.166, se = 0.032, t = −5.100, *p* < 0.00). Furthermore, in the fourth age group, the main effects were found for the modality (β = −0.094, se = 0.016, t = −5.718, *p* < 0.00) and lexical frequency variables (β = −0.074, se = 0.011, t = −6.495, *p* < 0.00), indicating that lexical recognition modality (as opposed to retrieval) resulted in faster responses and high-frequency words had lower RT compared to low-frequency words. A secondary interaction effect was observed for the 60–69 group with lexical frequency, indicating that this group experienced greater facilitation for high- and low-frequency words than the fourth age group, although high-frequency lexical words continued to elicit faster responses than low-frequency words (see Figure 1). Finally, a secondary interaction effect between modality and frequency was observed for the fourth age group and for both third age groups, demonstrating that lexical recognition always resulted in faster responses than retrieval during the different stages of aging (although this difference gradually narrows as age increases) and that high-frequency words facilitated responses compared to low-frequency words, regardless of the evaluated lexical modality (see Figure 1).

**Figure 2:**
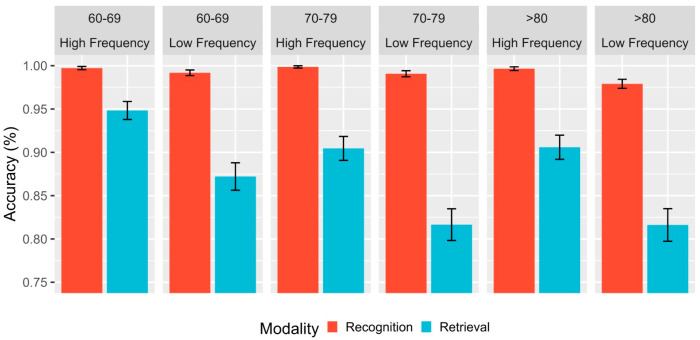
Mean accuracy (%) as a function of the age range (80+, vs. 60–69 vs. 70–79), modality (recognition vs. retrieval), and lexical frequency (high vs. low). Error bars represent within-subject adjusted 95% confidence intervals.

Furthermore, Table 3 presents the results of the generalized linear regression analysis, which indicate a significant main effect between age and accuracy. Specifically, individuals aged 80 years and older exhibited significantly lower accuracy in their responses than their younger counterparts (group 60–69: β = 0.667, se = 0.245, z = 2.729, *p* = 0.006; group 70–79: β = 0.547, se = 0.265, z = 2.068, *p* = 0.039). Moreover, the performance of individuals in the fourth age group revealed significant effects of modality and frequency factors, with a higher accuracy observed for recognition modality than for lexical retrieval (β = 1.616, se = 0.155, z = 10.429, *p* < 0.00). Additionally, high-frequency words were recognized with higher accuracy than low-frequency words (β = 0.811, se = 0.151, z = 5.359, *p* < 0.00; refer to Figure 2). Furthermore, a secondary interaction effect was observed between the 70–79 years group and modality, indicating that the differences between lexical recognition and retrieval were accentuated for this group, similar to what was observed in the fourth age group (refer to Figure 2). Finally, the regression model did not reveal any interaction effects for individuals aged 60–69 and 70–79, with the different variables evaluated due to the ceiling effect generated by the high accuracy rates in word recognition (above 99%) observed in these age groups (refer to Figure 2). However, a marginal effect was observed in the 60–69 group for modality and frequency (*p* = 0.069), which could be detected by increasing the sample size.

### 3.3. Results by Experimental Tasks

In the comparative analysis of experimental tasks between the third and fourth age groups, the linear mixed-effects regression (Table 4) revealed a significant main effect of age on RT. Specifically, the responses of the fourth age group were significantly slower compared to both third age groups (60–69 group: β = −0.285, se = 0.040, t = −7.050, *p*< 0.00; 70–79 group: β = −0.139, se = 0.040, t = −3.449, *p* = 0.001). Moreover, in the fourth age group, the main effects were found on naming (β = −0.271, se = 0.040, t = −6.717, *p* < 0.00) and priming (β = −0.311, se = 0.032, t = −9.672, *p* < 0.00) experiments, with significantly faster responses compared to the picture naming task (which had the highest RT). Similarly, a main effect was observed for the lexical frequency variable (β = −0.032, se = 0.015, t = −2.216, *p* = 0.027) in the fourth age group, where high-frequency words facilitated lexical access in comparison to low-frequency words.

**Figure 3:**
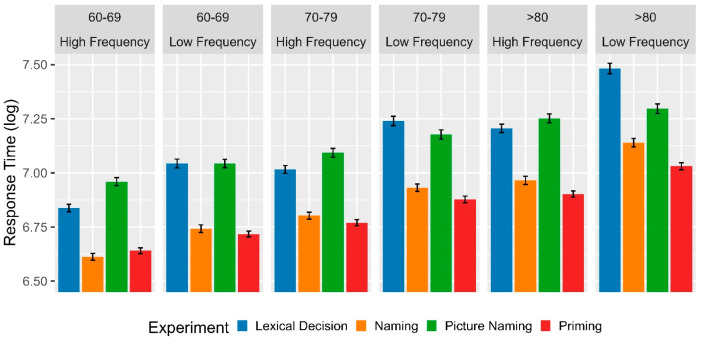
Mean log-transformed RT as a function of the age range (80+, vs. 60–69 vs. 70–79), experiment (lexical decision vs. naming vs. picture naming vs. priming), and lexical frequency (high vs. low). Error bars represent within-subject adjusted 95% confidence intervals.

Furthermore, the regression analysis showed significant interaction effects (secondary) between the groups 60–69 and 70–79 years with LDT, respectively. In both third age groups, this task generated greater facilitation (lower RT) compared to what was observed in the fourth age group (see Figure 3). Additionally, secondary interaction effects were observed for the fourth age group between LDT and naming with lexical frequency. This effect was significant for both high- and low-frequency words but with greater facilitation for high-frequency words. The analysis also showed interaction effects between each of the experimental tasks and lexical frequency for the 60–69 and 70–79 age groups, respectively. This supports that, in each of the experiments, high-frequency words exhibited lower RT compared to low-frequency words. On the other hand, this interaction demonstrates that all tasks generated significantly faster responses (lower RT) in the third age groups compared to the responses of the fourth age (see Figure 3). Finally, marginal effects were observed between the 60–69 group and the difference between picture naming and naming task (*p* = 0.071) and, in addition, between the 60–69 and frequency (*p* = 0.075) and 70–79 and frequency (*p* = 0.059) groups, which could probably become reliable with a larger sample size.

**Figure 4:**
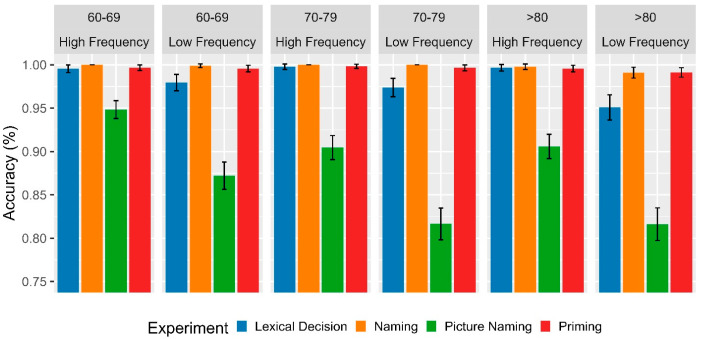
Mean accuracy (%) as a function of age range (80+ vs. 60–69 vs. 70–79), experiment (lexical decision vs. naming vs. picture naming vs. priming) and lexical frequency (high vs. low). Error bars represent within-subject adjusted 95% confidence intervals.

Regarding accuracy per experimental task, the generalized linear regression (Table 5) revealed a main effect between the fourth age group and the 60–69 age group (β = 0.662, se = 0.190, z = 3.482, *p* < 0.00) but not with the 70–79 age group. This suggests that 80 year olds made more errors than their younger peers but not compared to those in the intermediate age range (whose accuracy rates were similar). The fourth age group also showed the main effects for the LDT (β = 3.356, se = 0.525, z = 6.395, *p* < 0.00), naming (β = 6.103, se = 1.580, z = 3.864, *p* < 0.00), and priming (β = 5.185, se = 0.882, z = 5.879, *p* < 0.00), with more accurate responses and fewer errors than in the picture naming task. On the other hand, the picture naming task generated a higher number of errors in the older group throughout the entire age range considered in the study (see Figure 4). Additionally, a (secondary) interaction effect was found between the LDT and lexical frequency in participants older than 80 years. High-frequency words resulted in greater accuracy compared to low-frequency words in that task but not in the naming and priming experiments, where high accuracy rates were obtained for both conditions. Finally, the regression model showed no interaction effects for those aged 60–69 and 70–79 with the different variables evaluated due to the ceiling effect generated by the high accuracy rates (above 95%) in the LDT, naming, and priming tasks in these age groups (see Figure 4). Finally, a marginal effect was observed in the 60–69 group, LDT, and frequency (*p* = 0.090), which could be detected by increasing the sample size.

## 4. Discussion

The aim of this study was to investigate lexical access restrictions in people aged 80 years and older in terms of word recognition and retrieval modalities, as well as the type of task employed. To accomplish this, we compared the response time (RT) and accuracy of a fourth age group and two third age groups in three lexical recognition tasks (lexical decision task, naming, and priming) and one retrieval task (picture naming). The results show that the fourth age group has slower lexical access than both third age groups in terms of RT. Additionally, word retrieval modality continues to generate more difficulties in access (higher RT) than recognition modality. The picture naming task (retrieval) also generated slower responses than naming and priming (both recognition) in the advanced aging group. In terms of accuracy, the fourth age group made significantly more errors than their younger peers. Furthermore, the lexical retrieval modality generated greater difficulties (reduced accuracy) compared to the recognition modality. Specifically, during the fourth age, the picture naming task showed significantly lower accuracy compared to the good performance observed in the naming and priming tasks. Lastly, the results of the lexical frequency analysis confirm that high-frequency words are associated with lower RT and higher accuracy in all the tasks applied in the fourth age group. This supports the idea that the facilitation or inhibition effects generated by this variable may remain stable even beyond the age of 80 years.

Regarding the main findings, the results indicate a significant decrease in lexical access after the age of 80, both in recognition and retrieval, regardless of the task. This effect was predictable and can be attributed to well-known physiological changes that occur in advanced aging [3,6,7]. For instance, there is evidence that systematic reductions in neural circuitry, neurotransmitter reuptake, and axonal demyelination are primarily responsible for the reduction in the overall cognitive processing speed in old age [34]. Therefore, such changes could impede lexical access throughout the aging process, particularly during the fourth age, by slowing lexical processes of activation and competitor selection during word recognition or retrieval.

At the neuropsychological level, the significant increase in RT (response time) of lexical access during the fourth age could be attributed to the progressive decline of fluid intelligence during adulthood. Although no specific fluid intelligence and crystallized intelligence tests were taken in the study sample, the asymmetric decline of these two types of intelligence is well established in this population [8]. For example, various studies report that deficits in fluid intelligence have led to a decline in abstract thinking abilities, task planning, and mental quickness [4]. This decline is more pronounced post 80 years old [2,6]. Therefore, the increase in the RT of lexical access in advanced aging could occur because fluid intelligence declines abruptly during this stage of life [3]. This, along with the generalized loss of cognitive functionality [7], could result in a substantial decrease in the speed to efficiently solve a task [6]. Consequently, this negatively impacts the ability to activate lexical competitors and select from the representation corresponding to the sensory input (lexical recognition), as well as the ability to select the lemma corresponding to the conceptualized idea and its subsequent phonological encoding (lexical retrieval). In summary, it appears that deficits in fluid intelligence beyond the age of 80 years could strongly affect lexical access, leading to cognitive constraints that act independently of whether it is a matter of understanding a word or producing it.

Another significant effect identified is that, as people age, and particularly during the fourth age, they experience greater difficulties with lexical retrieval compared to recognition. Specifically, older individuals take significantly longer (higher RT) to select a specific word from their mental lexicon, phonologically encode it, and articulate it. The studies by Abrams and Farrell [18], Goral et al. [13], and Ouyang et al. [14], among others, support this finding: “linguistic aging” is dominated by word production difficulties [9,10,11,13,17]. Specifically, our results are consistent with the difficulties described in retrieving specific words during aging (proper names, labeling definitions, and lexical fluency [13,17,18]), although in our case, it was with common words/pictures. Also, our findings are consistent with the better performance observed in the word recognition modality [8,19], both RT and accuracy. Retrieval word difficulties are largely explained by transmission deficits that delay phonological activation [35], as well as the lack of inhibition of lexical competitors that block the retrieval and/or encoding of the target word [36].

According to the present study’s findings, this condition is accentuated after the age of 80. Additionally, we hypothesize that the greater number of representational levels involved in retrieval processes—activating multiple representations, selecting one among several competitors, phonologically encoding the word, gathering its phonemes, and generating the motor engram to articulate it—requires more cognitive resources than those required in recognition processes (activating competitors and selecting), which could also partially justify the higher response time generated by lexical retrieval.

When comparing the different experimental tasks, the priming and naming tasks (recognition) showed a significantly lower RT in the fourth age group compared to picture naming (retrieval). However, the LDT, which is a recognition task, did not show significant differences with the picture naming task and even generated a similar RT (see Table 1). What factors can explain adequate performances in the naming and priming tasks during advanced aging, and why does LDT generate higher TR?

Regarding naming and LDT, the work of Andrews [37] and Guzman [38] helped to explain such performances. Both authors claimed that the naming task eliminates the cognitive decisional factor of answering “yes or no” in the presence of a sequence of letters, thereby decreasing the cognitive accessory effects of the task and reducing the RT. For Ballota and Chumbley [39], the cognitive decisional factor in young people increased by 300 to 400 ms, the “original” RT involved in recognizing a lexical entry; from this, we infer that this value should be higher for elderly people. On the other hand, the priming task showed fast responses as a result of the powerful facilitating effect generated by the semantic pre-activation of a prime on the target word [21], which seems to improve recognition and reduce the RT, even during the fourth age.

As for the picture naming task, the marked increase in RT during advanced aging is explained by the continued decrease in fluid intelligence skills [12,40]. This condition, combined with the weakening of neural connections between lexical and phonological nodes [14], would be responsible for cortical activation not reaching the power required to achieve information transfer [34]. We believe that the systematic nature of these deficits could explain why people aged 80 and older are slower to produce words compared to their young peers.

Regarding accuracy, the results showed dissimilar behavior. On the one hand, when comparing the level of accuracy for all tasks applied between the third and fourth age groups, the group aged 80 and over had significantly lower accuracy, especially compared to the group aged 60–69 (see Table 5). However, in some recognition tasks like naming and priming, the level of accuracy remained stable in the fourth age group, and a ceiling effect was even observed in these tasks (see Table 1). This good performance declined when the task involved making a decision (LDT), where the older fourth age group made significantly more errors than their younger peers. Similarly, when the task required retrieving a specific item from the mental lexicon (picture naming), a noticeable increase in the error rate was evident in both the third and fourth age groups. This demonstrates that, under cognitively healthy conditions, when the task is “simple” and only requires recognizing (but not retrieving) specific words, the level of accuracy can remain stable throughout the life cycle, even in advanced stages of the life cycle [5,12].

Specifically, for the high accuracy rate obtained in some word recognition tasks (naming and priming), Ratcliff et al. proposed that persons of advanced aging seem to act more cautiously when visually recognizing words, which would significantly increase the response time (higher RT) but, at the same time, increase the recognition accuracy (i.e., 98% overall accuracy in the naming and priming tasks). Therefore, according to Ratcliff et al., for this lexical modality, the principle “the slower, the safer” would apply. In contrast, this cautious behavior described for word recognition does not seem to operate when the task consists of selecting a specific lexical item and encoding it phonologically (i.e., picture naming), since the higher transmission costs between semantics and phonology seem to significantly affect both the speed of processing (higher RT) and the accuracy (lower accuracy rate) of retrieval; thus, in word production tasks, “slower responses does not ensure higher accuracy”.

What explains the suitable accuracy performance on some recognition tasks but not on lexical retrieval? We assume that the excellent accuracy observed in “simpler” tasks would be associated with the maintenance of semantic abilities during aging [16]. These abilities function autonomously to the speed with which the lexicon is accessed. Different studies have confirmed that older people generally present a good conceptual performance, allowing them to access words with good accuracy [8]. Therefore, the results confirm that, regardless of the cognitive changes typical of the fourth age, responsible for the marked contrast between the decline of certain skills and the maintenance of others [2,3], crystallized intelligence remains stable [5] and can reinforce the accuracy of the process. This confirms the idea that word recognition and retrieval errors would reside in the operational abilities to lexical access and not in knowledge. However, the benefits associated with crystallized intelligence in advanced stages of old age would be reduced when the task involves higher processing costs, such as incorporating a decisional factor (LDT). During LDT, the ability to make a decision occurs after cue recognition [38,39], and at the same time, it could activate post-lexical semantic information derived from the target word, expanding the number of cognitive resources deployed to respond to the task. Therefore, it is plausible to assume that LDT has a negative impact on recognition accuracy, especially during the fourth age.

In turn, the benefits of crystallized intelligence could be diminished by the transmission deficit between lexical and phonological nodes (in the case of picture naming). Specifically, when a lexical node is activated, such semantic activation will spread and transmit to its corresponding phonological nodes. As a result, the phonological nodes will be activated, and the word will be produced [14]. The weakening of these connections in the advanced stages of aging would be responsible not only for delaying word production but also for reducing the benefits of crystallized intelligence by increasing the error rate.

Furthermore, all experiments showed that modulation of the response time (RT) and accuracy was directly associated with the lexical frequency variable. Specifically, lexical access facilitation (lower RT and higher accuracy) was observed for high-frequency words, while inhibition was observed for low-frequency words. These findings confirm the stability of lexical frequency as a robust predictor for word recognition and retrieval throughout the lifespan, including advanced aging. As mentioned in the Introduction, the lexical frequency effect is explained by the differing levels of semantic representation and familiarity presented by words, which impact the speed and accuracy of the processing [18,26]. Moreover, our data show that the difference in RT between low- and high-frequency words increases exponentially with aging in all experiments (see Table 1). This suggests that individuals in the fourth age may experience stronger cognitive changes relative to the earlier stages of aging [2].

The findings reported in the present study may have several implications and projections. First, at the theoretical level, they allow confirmation of the evolution of cognitive changes characteristic of the fourth age. For example, the findings would support that, during the fourth age, there would be a marked loss in fluid intelligence [2,3,4], reflected in the significant increase of TR in both lexical modalities and in the different tasks used. On the contrary, the maintenance of crystallized intelligence in advanced aging [5,6] would support the better performance observed in the accuracy rate (mainly in recognition tasks of low cognitive demand). From a psycholinguist, the findings confirmed that, during the fourth age, the transmission deficit between semantic and phonological nodes [41] would significantly increase, which would explain that cortical activation is not fast enough during word retrieval [35]. However, our findings do not allow us to rule out that the higher lexical processing costs observed in the group aged 80 years and older are also a consequence of the significant reduction of phonological activation [42] or due to an accentuated deficit of inhibition of lexical competitors [36].

At the clinical level, these findings are preliminary evidence that encourages the development of linguistic–cognitive intervention programs for people with advanced aging. Specifically, there would be a need for people aged 80 years and older to permanently train word retrieval skills such as naming objects, naming famous people or historical characters, verbal fluency, and labeling definitions (among others), favoring the use of lexical material that provides a greater cognitive challenge (i.e., words of low lexical frequency). Additionally, knowing lexical deficits can help psychologists and speech therapists to visualize more effective communication strategies for very old people (i.e., recovery and use of highly functional words). This is especially relevant in health and social care settings, where clear and effective communication directly impacts social participation, interaction with others, and overall life satisfaction. On the other hand, as projections, recognizing statistically normal linguistic changes during advanced aging would make it possible to distinguish them from those that are not, which would hypothetically help to identify complementary markers of cognitive impairment or mild dementia. In addition, the fact that there are people in very advanced stages of the life cycle with good lexical performance constitutes a source of data for the design of a possible model of resistance to neurodegenerative diseases, which would allow finding new protective factors that could be of great interest to the scientific community.

On the other hand, it should be noted that, in the present study, we focused exclusively on the changes in lexical processing related to advanced aging and controlled extensively for other factors that have been shown to positively or negatively influence lexical access, such as the person’s educational level, cognitive potential, emotional state, and reading comprehension [25]. For this reason, a process of homogenization and control of extralinguistic covariates was carried out on the entire study sample, which included the application of strict inclusion and exclusion criteria (i.e., educational level, cognitive status, emotional state, and reading comprehension). Controlling for these covariates allows us to propose that the findings presented in this study could be largely explained by the effect of advanced aging. However, it is not possible to rule out that individual differences in the above or other covariates (e.g., high sociocultural level) may have influenced the lexical skills observed in the third or fourth age groups.

Finally, it is necessary to clarify that the generalization of these findings should be taken with caution, considering that differences in cognitive performance between octogenarians, nonagenarians, and centenarians have been described [3,7], where people older than 90 years may present greater deficits in cognitive processes of short- and long-term memory and attention and executive functions, among others [1,3,7]. Therefore, although the fourth age has been defined as the age group representative of the final phase of the life cycle, it cannot be ignored that the lexical restrictions observed in our study group could increase in nonagenarian and centenarian persons (we do not know whether progressively or abruptly).

## 5. Conclusions

This study provides a better understanding of how lexical access abilities are restricted during advanced aging. The cognitive decline that characterizes this stage of life can affect the fast and accurate recognition and retrieval of words. People aged 80 years and older exhibited a strong constraint on lexical access, as they were slower and less accurate in recognizing and retrieving words than their younger peers. This restriction is deeper for the word retrieval modality, especially in the picture naming task, generating a higher response time (RT) and lower accuracy throughout aging, with significant effects in the fourth age.

The variable lexical frequency was also tested, and it seems to be a robust predictor of lexical recognition and retrieval throughout the life cycle. The deterioration of fluid intelligence and the reduction of internode transmission would reduce the ability to recognize and retrieve words, deficits that become more acute after the age of 80, impacting the speed of access and the accuracy rate. On the other hand, this study supports the idea that crystallized intelligence could reinforce the accuracy of lexical access during advanced aging, specifically in word recognition, although this would depend on the cognitive cost associated with the applied task.

## 6. Limitations

In terms of future research and limitations, these findings provide only initial empirical evidence on lexical access and its association with the cognitive decline characteristic of advanced aging. It is recommended that future research explore word retrieval during the fourth age, which is the most affected modality, to expand our understanding of how this age group experiences linguistic and cognitive changes. As a limitation, the present research only investigated lexical access through the visual modality in all experiments. This leaves the question of whether these findings would transfer to the auditory modality unanswered. Additionally, only isolated word processing was considered, not sentences or other types of contexts, which may affect lexical recognition and retrieval during advanced aging. Therefore, it is suggested that future research address these points.

## Figures and Tables

**Table 1:** RT (ms) and accuracy (%) by age group, lexical task, and lexical frequency.

	RT /HF	RT /LF	RT Overall	ACC/HF	ACC/LF	ACC Overall
LDT						
60–69	969.39	1204.34	1086.86	99	98	99
70–79	1157.15	1467.57	1312.36	99	98	99
80–92	1424.76	1920.99	1672.87	99	94	97
Naming						
60–69	769.58	881.46	825.52	100	99	100
70–79	927.93	1058.59	993.26	100	100	100
80–92	1114.41	1327.95	1221.18	98	97	98
Priming						
60–69	792.48	856.40	824.44	99	98	99
70–79	1353.05	1004.84	1178.94	99	98	99
80–92	1555.56	1185.14	1370.35	98	97	98
Picture Naming						
60–69	1141.79	1233.67	1187.73	95	86	91
70–79	1317.06	1446.51	1381.78	90	82	86
80–92	1610.07	1656.06	1633.06	90	82	86

ACC = accuracy; HF = high frequency; LF = low frequency.

**Table 2:** Mixed linear regression for the variables of age group, modality, and lexical frequency on RT (log).

	Estimate	se	t	Pr(>|t|)	
Intercept (fourth age)	7.177	0.025	284.629	0.000	***
Group 60–69	−0.329	0.033	−10.098	0.000	***
Group 70–79	−0.166	0.032	−5.100	0.000	***
Modality	−0.094	0.016	−5.718	0.000	***
Frequency	−0.074	0.011	−6.495	0.000	***
Group 60–69: Modality	−0.031	0.018	−1.669	0.099	
Group 70–79: Modality	−0.019	0.018	−1.043	0.300	
Group 60–69: Frequency	0.014	0.007	2.063	0.042	*
Group 70–79: Frequency	0.008	0.006	1.187	0.238	
Modality: Frequency	−0.029	0.011	−2.697	0.007	**
Group 60–69: Modality: Frequency	0.021	0.006	3.379	0.001	**
Group 70–79: Modality: Frequency	0.016	0.006	2.855	0.005	**

*** = *p* < 0.001; ** = *p* < 0.01; * = *p* < 0.05.

**Table 3:** Generalized linear regression for the variables of age group, modality, and lexical frequency on the accuracy (%).

	Estimate	se	z	Pr(>|z|)	
Intercept (fourth age)	4.797	0.208	23.055	0.000	***
Group 60–69	0.667	0.245	2.729	0.006	**
Group 70–79	0.547	0.265	2.068	0.039	*
Modality	1.616	0.155	10.429	0.000	***
Frequency	0.811	0.151	5.359	0.000	***
Group 60–69: Modality	−0.002	0.175	−0.014	0.989	
Group 70–79: Modality	0.426	0.191	2.228	0.026	*
Group 60–69: Frequency	−0.220	0.175	−1.255	0.210	
Group 70–79: Frequency	−0.022	0.205	−0.106	0.916	
Modality: Frequency	0.135	0.134	1.009	0.313	
Group 60–69: Modality: Frequency	−0.263	0.145	−1.817	0.069	
Group 70–79: Modality: Frequency	0.014	0.165	0.083	0.934	

*** = *p* < 0.001; ** = *p* < 0.01; * = *p* < 0.05.

**Table 4:** Mixed linear regression for the variables of age group, experiment type, and lexical frequency on RT (Log).

	Estimate	se	t	Pr(>|t|)	
Intercept (fourth age-picture naming)	7.306	0.032	231.750	0.000	***
Group 60–69	−0.285	0.040	−7.050	0.000	***
Group 70–79	−0.139	0.040	−3.449	0.001	***
LDT	0.037	0.035	1.048	0.296	
Naming	−0.271	0.040	−6.717	0.000	***
Priming	−0.311	0.032	−9.672	0.000	***
Frequency	−0.032	0.015	−2.216	0.027	*
Group 60–69: LDT	−0.127	0.042	−3.015	0.003	**
Group 60–69: Naming	−0.091	0.050	−1.830	0.071	
Group 60–69: Priming	−0.003	0.037	−0.077	0.939	
Group 70–79: LDT	−0.085	0.042	−2.032	0.045	*
Group 70–79: Naming	−0.048	0.049	−0.974	0.333	
Group 70–79: Priming	−0.005	0.037	−0.122	0.903	
Group 60–69: Frequency	−0.015	0.009	−1.791	0.075	
Group 70–79: Frequency	−0.015	0.008	−1.898	0.059	
LDT: Frequency	−0.118	0.022	−5.406	0.000	***
Naming: Frequency	−0.057	0.022	−2.604	0.010	**
Priming: Frequency	−0.027	0.020	−1.379	0.169	
Group 60–69: LDT: Frequency	0.057	0.015	3.781	0.000	***
Group 60–69: Naming: Frequency	0.037	0.012	3.103	0.002	**
Group 60–69: Priming: Frequency	0.042	0.011	3.853	0.000	***
Group 70–79: LDT: Frequency	0.048	0.014	3.328	0.001	**
Group 70–79: Naming: Frequency	0.039	0.011	3.505	0.001	***
Group 70–79: Priming: Frequency	0.025	0.010	2.500	0.012	*

*** = *p* < 0.001; ** = *p* < 0.01; * = *p* < 0.05.

**Table 5:** Generalized regression analysis for the variables of age group, experiment type, and lexical frequency on accuracy (%).

	Estimate	se	z	Pr(>|z|)	
Intercept (fourth age-picture naming)	2.619	0.193	13.571	0.000	***
Group 60–69	0.662	0.190	3.482	0.000	***
Group 70–79	0.001	0.182	0.004	0.997	
LDT	3.356	0.525	6.395	0.000	***
Naming	6.103	1.580	3.864	0.000	***
Priming	5.185	0.882	5.879	0.000	***
Frequency	0.618	0.153	4.033	0.000	***
Group 60–69: LDT	−0.236	0.558	−0.422	0.673	
Group 60–69: naming	7.332	15.122	0.485	0.628	
Group 60–69: Priming	−0.253	0.805	−0.314	0.754	
Group 70–79: LDT	0.599	0.608	0.985	0.324	
Group 70–79: Naming	15.078	58.535	0.258	0.797	
Group 70–79: Priming	0.744	0.855	0.871	0.384	
Group 60–69: Frequency	0.114	0.101	1.130	0.259	
Group 70–79: Frequency	−0.035	0.088	−0.400	0.689	
LDT: Frequency	0.972	0.463	2.099	0.036	*
Naming: Frequency	−0.166	0.755	−0.220	0.826	
Priming: Frequency	−0.212	0.455	−0.467	0.640	
Group 60–69: LDT: Frequency	−0.912	0.538	−1.695	0.090	
Group 60–69: Naming: Frequency	6.003	15.013	0.400	0.689	
Group 60–69: Priming: Frequency	−0.520	0.608	−0.855	0.392	
Group 70–79: LDT: Frequency	−0.182	0.588	−0.309	0.757	
Grupo 70–79: Naming: Frequency	−0.561	41.140	−0.014	0.989	
Grupo 70–79: Priming: Frequency	−0.098	0.636	−0.154	0.877	

*** = *p* < 0.001; * = *p* < 0.05.

## Data Availability

The data that support the findings of this study are available upon request from the corresponding author.

## Supplementary Materials

The following supporting information can be downloaded at https://www.mdpi.com/article/10.3390/brainsci13091343/s1. Supplementary Materials 1: List of words by Experimental Task; Supplementary Materials 2: Lexical Frequency (Espal); Supplementary Materials 3: Configuration of Experimental Trials.
