# Supplementary material for: Lexical Access Restrictions after the Age of 80
Source: Brain Sci. 2023 Sep 19;13(9):1343. doi: 10.3390/brainsci13091343 (PMC10526362; doi:10.3390/brainsci13091343)
Supplement: Supplementary file 1 [file brainsci-13-01343-s001.zip › brainsci-2564908-supplementary/Supplementary Materials/Supplementary Materials 1. List of words by Experimental Task.pdf]

List of words by experimental task

1.Lexical decision task: List of experimental words of high and low lexical frequency / high and low imaginability. Fillers and pseudowords are not included and were also not analyzed.

Words (2-3 syllable)	Lexical frequency (1=High, 0=Low)	Imaginability (1=High, 0=Low)
Agua	1	1
Niña	1	1
Manos	1	1
Cielo	1	1
Comida	1	1
Corazón	1	1
Cuerpo	1	1
Hija	1	1
Libro	1	1
Luna	1	1
Madera	1	1
Mujer	1	1
Parque	1	1
Médico	1	1
Verano	1	1
Capaz	1	0
Intención	1	0
Valores	1	0
Recurso	1	0
Razón	1	0
Real	1	0
Voluntad	1	0
Cierto	1	0
Sensación	1	0
Carácter	1	0
Conciencia	1	0
Causa	1	0
Lógica	1	0
Percepción	1	0
Moral	1	0
Bizcocho	0	1
Zorzal	0	1
Almeja	0	1

Maceta	0	1
Cubeta	0	1
Biombo	0	1
Molusco	0	1
Simio	0	1
Brasa	0	1
Madeja	0	1
Equino	0	1
Bisturí	0	1
Joroba	0	1
Betún	0	1
Morral	0	1
Cuantiosa	0	0
Conciso	0	0
Alarde	0	0
Morboso	0	0
Certera	0	0
Indaga	0	0
Agobio	0	0
Abruma	0	0
Sadismo	0	0
Pérfido	0	0
Melosa	0	0
Burdo	0	0
Fascina	0	0
Tortuoso	0	0
Sórdido	0	0

2. Naming task: List of experimental words of high and low lexical frequency / high and low positional syllable frequency (PSF) of the first syllable. Fillers and pseudowords are not included and were also not analyzed.

Words (2-3 syllable)	Lexical frequency (1=High, 0=Low)	PSF (1=High, 0=Low)
Comer	1	1
Abierto	1	1
Palabras	1	1
Local	1	1
Tomar	1	1
Militar	1	1
Médico	1	1
Música	1	1
Colegio	1	1
Cabeza	1	1
Casa	1	1
Ministro	1	1
Problema	1	1
Saber	1	1
Animal	1	1
Tribu	1	0
Bravo	1	0
Frases	1	0
Bronce	1	0
Montaña	1	0
Jardines	1	0
Barco	1	0
Olvidar	1	0
Castigo	1	0
Cría	1	0
Bruto	1	0
Trono	1	0
Crudo	1	0
Signo	1	0
Tregua	1	0
Calar	0	1
Nobel	0	1
Empuñar	0	1
Infectar	0	1

Distal	0	1
Pajar	0	1
Dilatar	0	1
Macaco	0	1
Encías	0	1
Codorniz	0	1
Mucosas	0	1
Rebatir	0	1
Tifus	0	1
Profesar	0	1
Sicario	0	1
Palpar	0	0
Polvorín	0	0
Baldío	0	0
Croquis	0	0
Clonar	0	0
Garfio	0	0
Factibles	0	0
Brotado	0	0
Captan	0	0
Balsero	0	0
Yerra	0	0
Rencillas	0	0
Bujías	0	0
Múltiplos	0	0
Bastidor	0	0

3. Priming task: List of experimental words of high and low lexical frequency / semantic-associative and ortho-phonologic prime. Fillers are not included and were also not analyzed.

High Frequency Words	Semantic/Associative prime
Doctor	Hospital
Cielo	Nubes
Planta	Flores
Venta	Negocio
Rural	Campo
Luna	Estrellas
Señor	Caballero
Copa	Vaso
Verdad	Sinceridad
Guerra	Combate
Diario	Revista
Reyes	Príncipe
Cuento	Novela
Viento	Tormenta
Lucha	Pelea
Amor	Lealtad
Abril	Junio
Arte	Escultura
Barrio	Población
Carne	Longaniza

Low Frequency Words	Semantic/Associative Prime
Fríjol	Poroto
Zurda	Izquierda
Panal	Colmena
Puercos	Cerdos
Albo	Blanco
Asar	Hornear
Palpar	Tocar
Helar	Enfriar
Cercar	Alambrar
Cojín	Almohada

Hurtar	Sustraer
Cirio	Candelabro
Diluir	Disolver
Hierve	Calienta
Vergel	Huerta
Betún	Lustrín
Costal	Saco
Pinchar	Punzar
Toser	Carraspear
Reúnen	Congregan

High Frequency Words	Ortho-phonologic Prime
Comer	Correr
Culpa	Culta
Terror	Tenor
Día	Tía
Cuenta	Cuenca
Japón	Jamón
Junto	Junco
Liga	Lija
Cuando	Cuanto
Banda	Banca
Hielo	Hierro
Llevar	Llenar
Renta	Renca
Blanco	Blando
Cuerpo	Cuerno
Marca	Marta
Falta	Falsa
Sala	Sana
Gana	Gala
Poco	Pozo

Low Frequency Words	Ortho-phonologic Prime
Topar	Tocar
Templar	Temblar

Atril	Abril
Parco	Parto
Surtir	Surgir
Jarrón	Jabón
Hurgar	Hurtar
Calar	Cavar
Pegué	Pequé
Bajón	Balón
Trompo	Trombo
Rajó	Rayó
Brincar	Brindar
Tienta	Tienda
Zarpa	Zarza
Lanas	Latas
Bancas	Bandas
Morral	Modal
Manchar	Mandar
Fuman	Fugan

4. Picture Naming task: List of experimental words of high and low lexical frequency / bysyllable, trisyllable and tetrasyllable. Fillers are not included and were also not analyzed.

Words	Frequency (1= High/ 0=Low)	Number of syllables
Avión	1	2
Hojas	1	2
Huesos	1	2
Huevos	1	2
Perro	1	2
Nariz	1	2
Nido	1	2
Niños	1	2
Puerta	1	2
Reloj	1	2
Tigre	1	2
León	1	2
Mapa	1	2
Radio	1	2
Arroz	1	2
Carta	1	2
Chino	1	2
Hielo	1	2
Indio	1	2
Reina	1	2
Botella	1	3
Anillo	1	3
Árboles	1	3
Bandera	1	3
Caballo	1	3
Cadena	1	3
Cocina	1	3
Familia	1	3
Guitarra	1	3
Antena	1	3
Oveja	1	3
Sombrero	1	3
Ventana	1	3
Aceite	1	3

Espejo	1	3
Zapatos	1	3
Monedas	1	3
Oreja	1	3
Ajedrez	1	3
Iglesia	1	3
Chocolate	1	4
Herramientas	1	4
Calendario	1	4
Presidente	1	4
Cementerio	1	4
Escalera	1	4
Teléfono	1	4
Edificio	1	4
Uniforme	1	4
Zapatero	1	4
Animales	1	4
Diccionario	1	4
Pescadores	1	4
Secretaria	1	4
Policía	1	4
Oficina	1	4
Batería	1	4
Cerámica	1	4
Futbolista	1	4
Televisor	1	4
Ají	0	2
Apio	0	2
Cebra	0	2
Gorros	0	2
Peras	0	2
Pito	0	2
Sartén	0	2
Carnet	0	2
Confort	0	2
Cojín	0	2
Brocha	0	2
Habas	0	2
Pinza	0	2

Grifos	0	2
Higos	0	2
Maní	0	2
Betún	0	2
Hippie	0	2
Pulpo	0	2
Yogur	0	2
Candado	0	3
Espuela	0	3
Durazno	0	3
Erizo	0	3
Estuche	0	3
Fósforos	0	3
Carrusel	0	3
Ardilla	0	3
Velador	0	3
Canguro	0	3
Pepino	0	3
Almejas	0	3
Pesebre	0	3
Bombones	0	3
Calzones	0	3
Champaña	0	3
Jarabe	0	3
Medusa	0	3
Medidor	0	3
Parlantes	0	3
Basurero	0	4
Espárragos	0	4
Herraduras	0	4
Carretilla	0	4
Ventilador	0	4
Mayonesa	0	4
Semáforo	0	4
Almohadas	0	4
Azafata	0	4
Mermelada	0	4
Neumático	0	4
Remolino	0	4

Servilleta	0	4
Urinario	0	4
Aceituna	0	4
Aspirina	0	4
Calcetines	0	4
Ensaladas	0	4
Candelabro	0	4
Orégano	0	4
