# Supplementary material for: Lexical Access Restrictions after the Age of 80
Source: Brain Sci. 2023 Sep 19;13(9):1343. doi: 10.3390/brainsci13091343 (PMC10526362; doi:10.3390/brainsci13091343)
Supplement: Supplementary file 1 [file brainsci-13-01343-s001.zip › brainsci-2564908-supplementary/Supplementary Materials/Supplementary Materials 2. Lexical Frequency (Espal).pdf]

LEXICAL FREQUENCY BY WORDS(ESPAL)

WORD	HIGH FREQUENCY	WORD	LOW FREQUENCY
abierto	68,085	abruma	0,601
abril	185,513	aceituna	1,283
aceite	25,427	agobio	0,747
agua	277,367	ají	1,147
ajedrez	14,205	alarde	1,566
amor	208,368	albo	0,666
anillo	23,693	almeja	0,884
animal	67,202	almejas	0,647
animales	118,048	almohadas	1,407
antena	19,144	apio	1,095
arroz	18,328	ardilla	1,372
arte	163,874	asar	0,777
avión	48,100	aspirina	1,001
banda	179,620	atril	0,673
bandera	48,091	azafata	1,062
barco	42,739	bajón	0,939
barrio	81,765	baldío	0,686
batería	35,052	balsero	0,926
blanco	127,861	bancas	0,718
botella	15,619	bastidor	0,773
bravo	16,766	basurero	0,799
bronce	30,159	betún	0,647
bruto	14,959	biombo	0,822
caballo	82,493	bisturí	0,754
cabeza	220,039	bizcocho	0,832
cadena	68,664	bombones	0,858

calendario	30,012	brasa	1,515
capaz	92,315	brincar	0,874
carne	78,870	brocha	0,617
carta	121,538	brotado	0,790
carácter	153,643	bujías	0,783
casa	403,590	burdo	0,871
castigo	26,230	calar	0,760
causa	174,502	calcetines	1,527
cementerio	23,095	calzones	1,465
cerámica	14,754	candado	1,066
chino	29,983	candelabro	0,952
chocolate	14,514	canguro	0,981
cielo	103,583	captan	0,796
cierto	212,664	carnet	1,322
cocina	38,593	carretilla	0,832
colegio	65,038	carrusel	0,975
comer	67,735	cebra	1,277
comida	49,939	cercar	0,621
conciencia	90,161	certera	0,991
copa	117,710	champaña	1,573
corazón	148,457	cirio	0,728
crudo	14,199	clonar	0,601
cría	14,244	codorniz	0,946
cuando	326,414	cojín	0,903
cuenta	322,721	conciso	0,981
cuento	30,480	confort	1,429
cuerpo	244,307	costal	0,939
culpa	42,366	croquis	0,777
diario	99,447	cuantiosa	0,751

diccionario	17,477	cubeta	0,881
doctor	67,270	dilatar	0,871
día	418,513	diluir	0,988
edificio	90,863	distal	0,997
escalera	22,751	durazno	1,387
espejo	26,273	empuñar	0,855
falta	193,412	encías	0,679
familia	277,153	ensaladas	1,322
frases	20,057	equino	0,835
futbolista	32,147	erizo	1,521
gana	38,119	espuela	0,832
guerra	346,495	espárragos	0,926
guitarra	53,887	estuche	1,477
herramienta	29,580	factibles	0,864
hielo	30,201	fascina	0,920
hija	125,053	fríjol	0,751
hojas	50,846	fuman	0,686
huesos	20,808	fósforos	1,108
huevos	23,716	garfio	0,767
iglesia	216,325	gorros	0,984
indio	20,408	grifos	0,725
intención	77,067	habas	1,538
japón	74,594	helar	0,689
jardines	18,582	herraduras	0,767
junto	344,800	hierve	0,955
león	102,423	higos	1,550
libro	210,867	hippie	1,030
liga	117,720	hurgar	0,653
llevar	120,959	hurtar	0,738

local	115,179	indaga	0,861
lucha	167,517	infectar	0,760
luna	67,641	jarabe	1,525
lógica	40,017	jarrón	0,952
madera	60,460	joroba	0,855
manos	197,591	lanas	0,890
mapa	23,813	macaco	0,689
marca	63,537	maceta	0,806
militar	175,714	madeja	0,816
ministro	241,090	manchar	0,738
monedas	22,221	maní	1,287
montaña	42,151	mayonesa	0,608
moral	68,232	medidor	0,702
mujer	287,992	medusa	1,423
médico	75,722	melosa	0,796
música	217,287	mermelada	1,079
nariz	20,281	molusco	0,617
nido	18,254	morboso	0,634
niña	38,005	morral	0,650
niños	115,975	mucosas	0,838
oficina	51,502	múltiplos	0,871
olvidar	34,698	neumático	1,410
oreja	21,012	nobel	1,279
oveja	20,925	orégano	0,773
palabras	216,182	pajar	0,621
parque	88,315	palpar	0,770
percepción	23,348	panal	0,936
perro	69,971	parco	0,796
pescadores	17,861	parlantes	0,939

planta	75,647	pegué	0,604
poco	112,684	pepino	1,518
policía	153,087	peras	1,512
presidente	412,261	pesebre	1,415
problema	220,286	pinchar	0,900
puerta	158,978	pinza	1,023
radio	104,080	pito	1,495
razón	212,192	polvorín	0,936
real	309,384	profesar	0,926
recurso	83,331	puercos	0,777
reina	92,949	pulpo	1,446
reloj	26,146	pérfido	0,816
renta	42,970	rajó	0,617
reyes	70,081	rebatir	0,881
rural	45,238	remolino	1,484
saber	169,190	rencillas	0,894
sala	108,603	reúnen	1,462
secretaria	17,500	sadismo	0,780
sensación	40,345	sartén	1,496
señor	49,052	semáforo	1,473
signo	27,546	servilleta	1,248
sombrero	21,272	sicario	0,608
televisor	33,782	simio	0,984
teléfono	33,664	surtir	0,864
terror	35,796	sórdido	0,783
tigre	26,474	templar	0,955
tomar	134,378	tienta	0,630
tregua	15,427	tifus	0,942
tribu	20,051	topar	0,624

trono	37,853	tortuoso	0,955
uniforme	30,532	toser	0,744
valores	107,043	trompo	0,660
venta	70,178	urinario	0,679
ventana	41,566	velador	0,627
verano	83,302	ventilador	1,144
verdad	202,744	vergel	0,871
viento	67,017	yerra	0,790
voluntad	125,164	yogur	1,017
zapatero	43,646	zarpa	0,692
zapatos	14,036	zorzal	0,611
árboles	52,903	zurda	0,838
X=	96,568	X=	0,950
