# Supplementary material for: Lexical Access Restrictions after the Age of 80
Source: Brain Sci. 2023 Sep 19;13(9):1343. doi: 10.3390/brainsci13091343 (PMC10526362; doi:10.3390/brainsci13091343)

Configuration of experimental trials for each lexical task

Lexical Decision Task

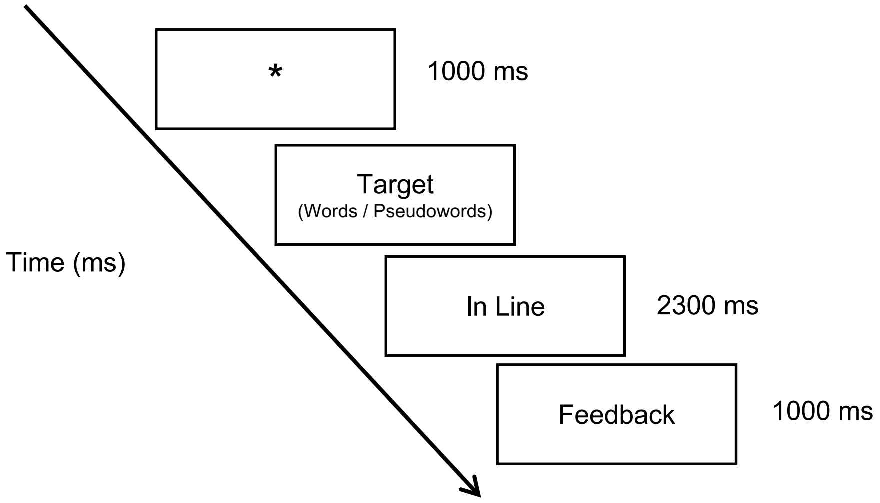

Naming Task

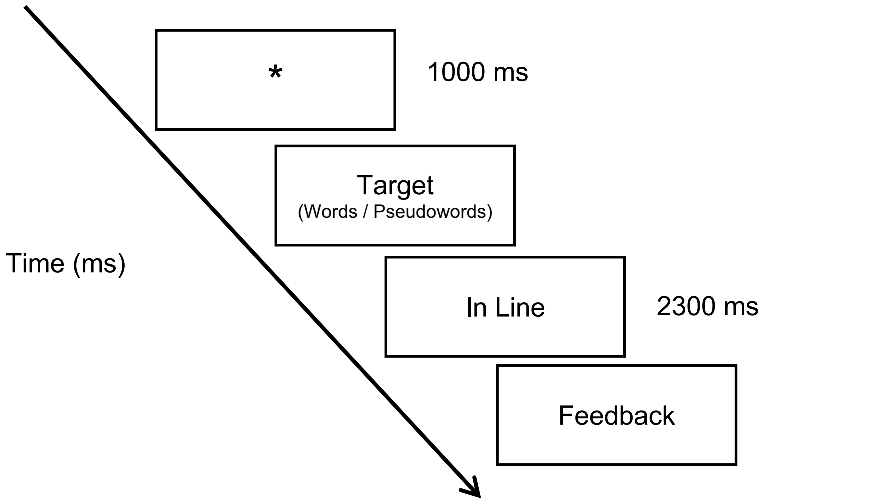

Priming Task

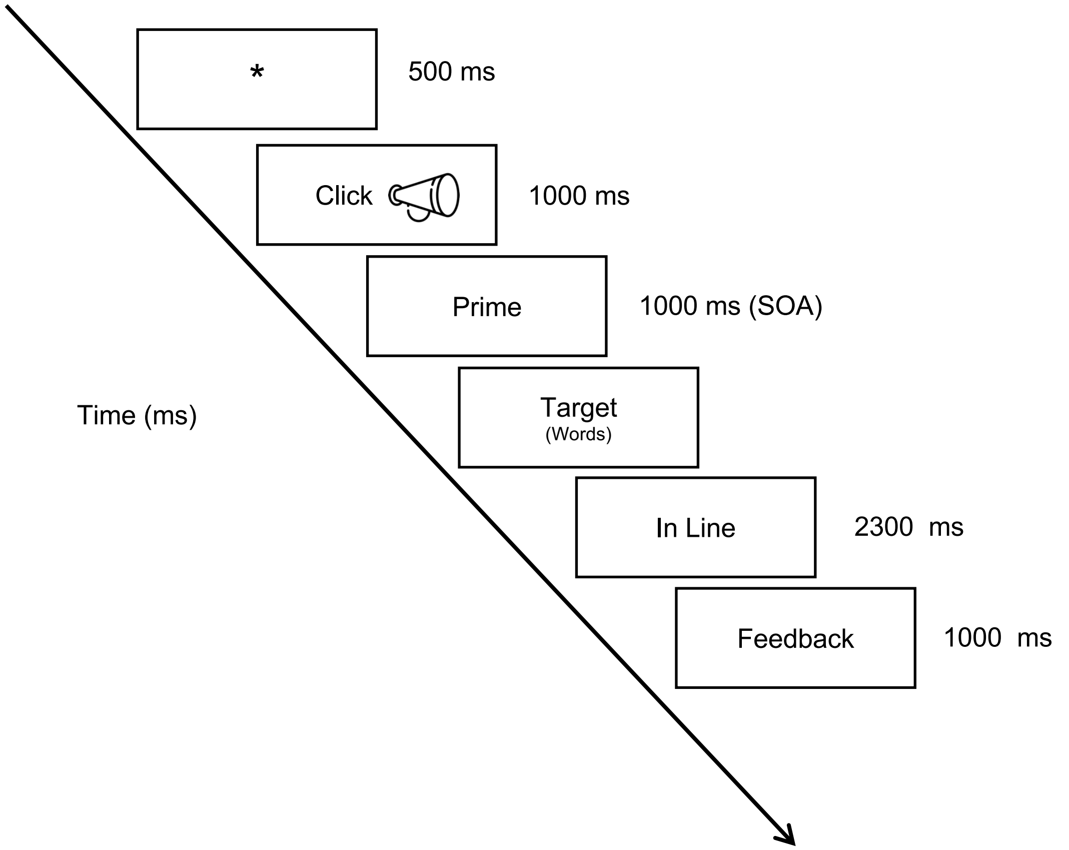

Picture Naming Task

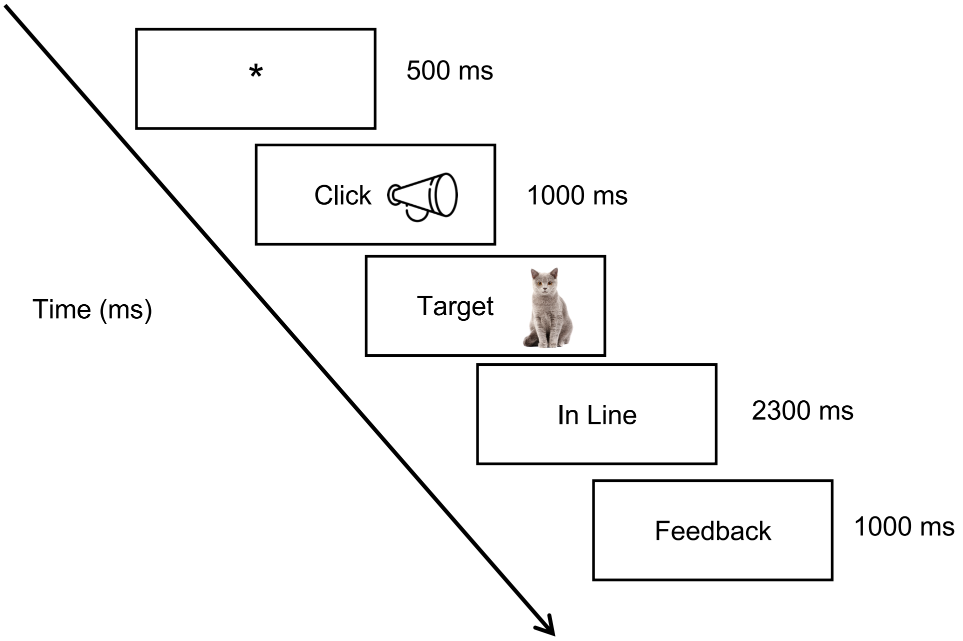

Supplement: Supplementary file 1 [file brainsci-13-01343-s001.zip › brainsci-2564908-supplementary/Supplementary Materials/Supplementary Materials 3. Configuration of Experimental Trials.pdf]
